# Synergistic Effect of Compounds from a Chinese Herb: Compatibility and Dose Optimization of Compounds from N-Butanol Extract of Ipomoea stolonifera

**DOI:** 10.1038/srep27014

**Published:** 2016-06-03

**Authors:** Congyi Cai, Yicun Chen, Shuping Zhong, Yanmei Zhang, Jiyang Jiang, Han Xu, Ganggang Shi

**Affiliations:** 1 Department of Pharmacology, Shantou University Medical College, Shantou 515041, Guangdong, China; 2 Department of Pharmacy, Second Affiliated Hospital, Shantou University Medical College, Shantou 515041, Guangdong, China; 3 Department of Biochemistry and Molecular Biology, Keck School of Medicine, University of Southern California, Los Angeles, California 90033, USA; 4 Department of Cardiovascular Diseases, First Affiliated Hospital, Shantou University Medical College, Shantou 515041, Guangdong, China

## Abstract

The herbal medicine *Ipomoea stolonifera* (*I. stolonifera*) has previously been shown to have considerable anti-inflammatory potential *in vivo and in vitro*. To establish a method for exploring the synergistic effects of multiple compounds, we study the compatibility and dose optimization of compounds isolated from n-butanol extract of *I. stolonifera* (BE-IS). Raw264.7 cell was treated with lipopolysaccharide (LPS) in the presence of compounds from BE-IS, namely scopoletin, umbelliferone, esculetin, hesperetin and curcumin, using the orthogonal design, uniform design and median-effect method. To verify the best efficacy of principal constituents *in vivo*, the uniform design was used in the croton oil-induced mouse ear edema model. The results from LPS-induced the production of prostaglandin E_2_ (PGE_2_) show that, esculetin, curcumin and hesperetin were the principal constituents that had synergistic effects when used at the optimal ratio. Additionally, the principal constituents were found to work synergistically in the croton oil-induced mouse ear edema model at low doses. It turned out that the three experimental optimization and analysis methods (orthogonal design, uniform design and median-effect method) can be effectively used to solve both compatibility and dose optimization for combined use of multiple compounds.

To enhance therapeutic efficacy and reduce adverse effects, practitioners of traditional Chinese medicine (TCM) prescribe a combination of plant species/minerals, called formulae, based on clinical experience. In modern times, formulae are regarded as a combination of multiple compounds used to treat a disease. In particular, for TCM, the term formulae are particularly appropriate because there are a great many of compounds in one herb. Formulae possess the advantage that, at least in some formulae, multiple components could hit multiple targets and exert synergistic therapeutic efficacies^1^,^2^.

Increasing evidence demonstrates that, in treating illnesses, including cancer^3^ and HIV/AIDS^4^, treatment regimens containing multiple drugs with distinct but related mechanisms can usually amplify the therapeutic efficacies of each agent, leading to maximal therapeutic efficacy with minimal adverse effects. The complexity of therapy suggests that treatment protocols should be carefully designed. However, it remains a puzzle as to how to construct and identify a prescription containing the best combination and optimal doses to combat diseases. To solve this question, we designed a series of methods to rapidly identify the compatibility and optimized doses of drugs in combination, and validated the methods using compounds isolated from n-butanol extract of *Ipomoea stolonifera* (BE-IS).

*Ipomoea stolonifera* (*I. stolonifera*), also known as fiddle-leaf morning-glory, is an herb from the Convolvulaceae family that has been traditionally used to treat inflammatory diseases in the Chaoshan area^5^. In traditional Chinese medicine, it is considered to have the effects of treating inflammatory disorders, and is typically used in the treatment of rheumatoid arthritis^6^. Early experiments indicated n-butanol extract from *I. stolonifera* (BE-IS) has considerable anti-inflammatory potential *in vivo and in vitro*, and a number of isolated compounds (i.e. scopoletin (Sco), umbelliferone (Umb), esculetin (Esc), hesperetin (Hes), and curcumin (Cur)) from BE-IS have demonstrated efficacy in treating inflammation^7^,^8^. However, use of single compounds from BE-IS dose not achieve high efficiency comparable to BE-IS. Therefore, the aim of the present study was to investigate the synergistic potential among these compounds, search for the best combination and determine their combined efficacy in treating inflammation.

Inflammation is the body’s immediate response of the immune system to infection and irritation^9^, playing key roles in pathogen clearance and wound healing^10^. The invasion of pathogens causes a series of cellular immune responses, in which macrophages are critically involved. Macrophages directly recognize pathogens, ingest them by phagocytosis and produce various pro-inflammatory mediators, including TNF-α, IL-1β, IL-6, NO and PGE_2_. These mediators play an essential role in not only strengthening the innate immune response, but also in activating other immune cells to promote a secondary immune response^10^. Thus, we used the model of LPS-induced the production of pro-inflammatory mediators in Raw264.7 cell.

The production of pro-inflammatory mediators needs to be restrained as excessive amounts of these molecules result in a chronic inflammatory state, which severely damages tissue and leads to the initiation of various inflammatory diseases, including inflammatory bowel diseases, metabolic diseases and arthritis^11^,^12^,^13^. Thus, agents that are effective at controlling these pro-inflammatory mediators are considered to be promising therapeutics for these diseases^14^, such as steroidal anti-inflammatory drugs (SAIDs) and nonsteroidal anti-inflammatory drugs (NSAIDs). However, prolonged use of these drugs may produce many adverse effects, including gastrointestinal disorders^15^, immunodeficiency and humoral disturbances^16^. So the optimized combination of the bioactive compounds with fewer adverse effects could be developed for long-term administration.

In this study, the following technical procedures were taken to investigate the compatibilities and optimized concentrations for combined use of compounds: screening principal constituents by orthogonal design, analyzing the optimal concentrations and best efficacy of principal constituents by uniform design, confirming synergies between principal constituents by the median-effect method, and analyzing the mechanisms of synergistic effects of the principal constituents. This optimization mode can be used in the study of other drug combinations, such as combined use of multiple anti-hypertension drugs for hypertension, glucocorticoid and β_2_ receptor agonists for asthma, and multiple antibiotics for bacterial infection.

Use of these three methods showed that Esc, Cur and Hes are the principal constituents having synergistic effects, when used at the optimal ratio, based on a reduction in LPS-induced the production of PGE_2_. Cur significantly reduces LPS-induced the production of NO and IL-1β, while Esc significantly reduces the production of IL-6. Consistent with these *in vitro* data, in the croton oil-induced mouse ear edema model, the principal constituents work synergistically at low doses. Then the results of verification experiments futher demonstrate the synergistic effects of the three principal constituents.

## Results

### Effect of compounds from BE-IS on the production of PGE_2_

**Figure 1:**
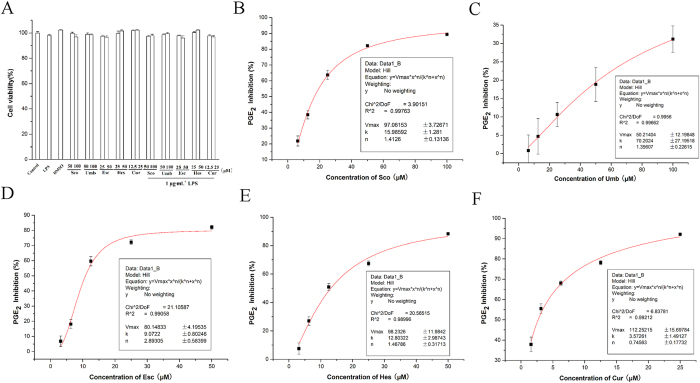
Concentration-effect relationship of compounds from BE-IS on LPS-induced the production of PGE_2_. (**A**) Cytotoxic effects of compounds from BE-IS. Raw264.7 cells were treated with Sco, Umb, Esc, Hes and Cur individully or followed by 1 μg·mL^−1^ LPS for 24 h. Cells were then subjected to MTT assay as described in the Methods. (**B–F**) Concentration-effect relationship of Sco (**B**), Umb (**C**), Esc (**D**), Hes (**E**) and Cur (**F**) on LPS-induced the production of PGE_2_. Drugs were dissolved in DMSO and diluted in DMEM to a final value of DMSO <0.1%. All values are presented as the mean ± S.E. and are the result of three independent experiments.

Consistent with previous data, Sco, Umb, Esc, Hes and Cur individual or followed by 1 μg·mL^−1^ LPS did not have any significant cytotoxic effects on Raw264.7 cells at the concentrations used in this study, as determined by MTT assay (Fig. 1). Five compounds exhibited an inhibitory effect on LPS-induced PGE_2_ release in a dose-dependent manner (Fig. 1). Using Origin7.5 software, the Hill equation of each compound from BE-IS was established, and the E_max_, k(IC_50_) and correlation coefficient R were obtained. As shown in Table 1, the Hill equation-fitted correlation coefficients were over 0.99 for Sco, Umb, Esc, Hes and Cur, indicating that the dose-efficacy relationship of each compound matched the Hill model.

### Screening principal constituents from BE-IS by orthogonal design

According to the effective concentration range of compounds from BE-IS, 4 different concentrations were chosen for them individually. The L_16_(4^5^) orthogonal design table (Table 2) was used to design an experiment comprising 16 combinations. Direct analysis and variance analysis were used to analyze the result, confirming the influence degree of each compound on the combinative effect.

In Table 2, K1, K2, K3, and K4 indicate the sum of the inhibition rates of each factor at various levels; avK1, avK2, avK3, and avK4 indicate the average inhibition rate of each factor at various levels; and R is the range of the average inhibition rate of each factor at various levels (range = maximum of average inhibition rate – minimum of average inhibition rate). It reflects the influence on the experimental result by variations in the level of each factor. According to R value and analysis of variance (Table 2), variation in the concentration of Esc had the greatest effect on the result (P < 0.01), followed by variations in the concentration of Cur (P < 0.01) and Hes (P < 0.05), respectively. Therefore, these were determined to be the principal constituents from BE-IS when used in combination.

### The optimal concentrations and best efficacy of principal constituent analysis by uniform design

**Figure 2:**
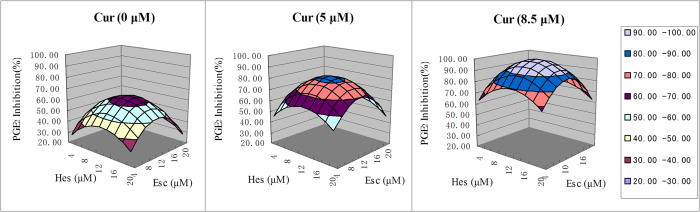
Response surface analysis and effect prediction of PGE_2_ inhibition by different combinations of principal constituents from BE-IS. The response surface figure was drawn with a fixed Cur, where Esc and Hes were used as the X and Y axis, respectively, and PGE_2_ release inhibition rate was used in the Z axis.

In this study, Esc, Cur and Hes were found to be the principal constituents by orthogonal design. Seven different concentrations were chosen for Esc, Cur and Hes, individually. The U_7_(7^3^) uniform design table (Table 3) was used to design an experiment of 7 combinations (Table 3). Esc, Cur and Hes were independent variables, and the PGE_2_ release inhibition rate was the dependent variable Y. Excel Solver tool was used to find the minimum values of the residual sum of squares between the experimental values and the predicted values, and a multivariate quadratic equation was established: Y = −0.3495 × E^2^ + 8.0821 × E + 0.1480 × C^2^ + 2.9069 × C − 0.2212 × H^2^ + 5.5140 × H − 16.7161. The data indicated that Esc and Hes had a quadratic response surface effect. Additionally, it was found that the efficacy of Cur increased in direct correlation to an increase in concentration. Because figures are not adequate to illustrate multi-dimensional surfaces, a response surface figure was drawn with a fixed Cur, where Esc and Hes were represented on the X and Y axis, respectively, and PGE_2_ release inhibition rate was represented by the Z axis (Fig. 2).

The above equation and response surface analysis predicted that the optimal concentrations were: Esc 11.6 μM, Cur 8.5 μM, and Hes 12.5 μM with an expected efficacy of 99.77%. Because this combination was not one of the seven combinations tested, this combination was further tested in parallel with combination 7, which was the combination with the best efficacy in the seven tested combinations. These two combinations showed similar results upon testing (Table 3), suggesting that our prediction was correct.

### Interaction results among principal constituents determined by median-effect method analysis

Each drug had 5 pairs of D and Fa (Table 4). Correlations of log-log coordinates were examined and the correlation coefficient R was calculated. Double logarithmic coordinates linear regression analysis was conducted by using equation (1), and m and D_50_ were calculated (Table 5). R was found to be 0.99 for each drug, suggesting that our experimental design was appropriate. When D_50_ and m were obtained, the function relation between dose D and inhibition rate Fa was established by using equation (1).

As shown in Table 5, the m values of Esc, Cur, and Hes, and combinations of Esc & Cur, Esc & Hes, and Cur & Hes were similar. Their double logarithm coordinate lines were almost parallel, indicating that the effects of these compounds were not independent, and therefore k = 0. Assuming Fa = a%, the combination index (CI)a was obtained by equations (1), (2), (3), and (4). As shown in Table 5, when combination of Esc & Cur and Cur & Hes were given at high or low dosages, CI < 1; when combination of Esc & Hes had Fa ≥ 30%, CI < 1.

### Effect of principal constituents from BE-IS on the production of NO and cytokines

**Figure 3:**
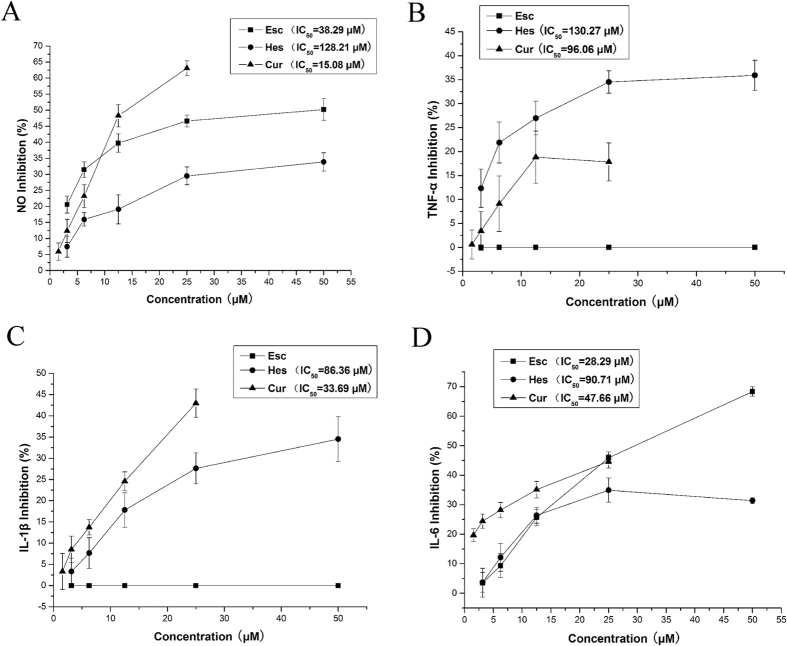
Effect of principal constituents from BE-IS on LPS-induced the production of NO and cytokines. Drugs were dissolved in DMSO and diluted in DMEM to a final value of DMSO <0.1%. All values are presented as the mean ± S.E. and are the result of three independent experiments.

The data indicated that Cur effectively inhibited LPS-induced the production of NO (IC_50_ = 15.08 μM) and IL-1β (IC_50_ = 33.69 μM), whereas Esc inhibited LPS-induced the production of IL-6 (IC_50_ = 28.29 μM), but had no effect on TNF-α or IL-1β. Additionally, Hes inhibited the production of NO, TNF-α, IL-1β and IL-6, but not significantly (Fig. 3).

### The optimal dosages and best efficacy of principal constituent analysis by uniform design *in vivo*

Seven different dosages were chosen for each of the principle components and the U_7_(7^3^) uniform design table (Table 6) was used to design an experiment of 7 combinations (Table 6). In Table 6, Esc, Cur, and Hes were independent variables, and inhibition of croton oil-induced mouse ear edema was the dependent variable Y. Excel Solver tool was used to find the minimum values of residual sum of squares between the experimental values and the predicted values, and a multivariate quadratic equation was established: Y = 0.000477 × E^2^ + 0.071445 × E − 0.01198 × C^2^ + 1.426827 × C − 0.00077 × H^2^ + 0.293119 × H − 49.0712.

The above equation and Excel Solver tool predicted that the optimal dosages were: Esc 337 mg·kg^−1^, Cur 60 mg·kg^−1^, and Hes 191 mg·kg^−1^. The expected efficacy was: 99.63%. Because this combination was not one of the 7 combinations tested, this combination was further tested in parallel with combination 6, which was the combination with the best efficacy in the seven tested combinations. These two combinations showed similar results upon testing (Table 6), suggesting that our prediction was correct. Results indicate that combination therapy may prove invaluable given the synergistic potential as well as the possibility of utilizing smaller dosages.

### Verification of the results

**Figure 4:**
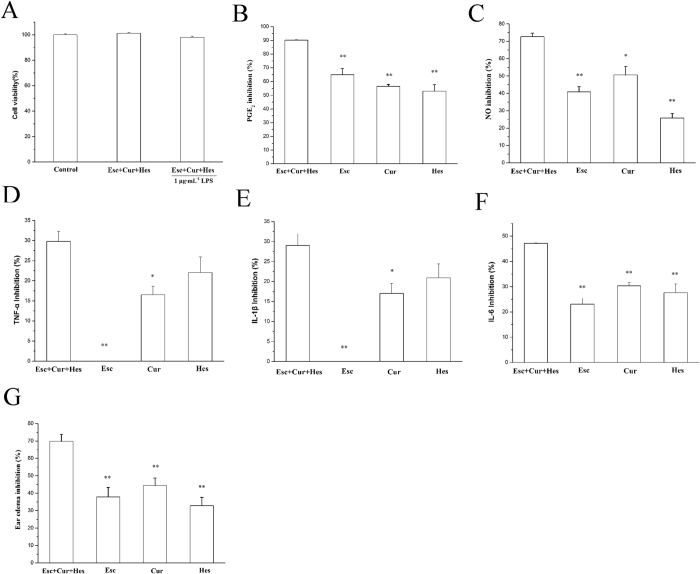
The inhibition on the production of inflammatory mediators, cytokines and ear edema after treatment with the combination and each compound of Esc, Cur and Her at the indicated optimal concentrations. (**A**) Cytotoxic effects of the combination of Esc, Cur and Her at the indicated optimal concentrations. Raw264.7 cells were treated with the combination of Esc 11.6 μM, Cur 8.5 μM, and Hes 12.5 μM alone or followed by 1 μg·mL^−1^ LPS for 24 h. Cells were then subjected to MTT assay as described in the Methods. (**B–F**) The inhibition on the production of PGE_2_ (**B**), NO (**C**), TNF-α (**D**), IL-1β (**E**) and IL-6 (**F**) after treatment with the combination and each compound of Esc, Cur and Her at the indicated optimal concentrations of 11.6 μM, 8.5 μM and 12.5 μM, respectively. (**G**) The inhibition on croton oil-induced mouse ear edema after treatment with the combination and each compound of Esc, Cur and Her at the indicated optimal dosage of 337 mg·kg^−1^, 60 mg·kg^−1^ and 191 mg·kg^−1^, respectively. All values are presented as the mean ± S.E. and are the result of three independent experiments. ^*^p < 0.05, ^**^p < 0.01 as compared to Esc+ Cur+Hes group. (Esc+ Cur+Hes represent combination of Esc, Cur and Hes).

To verify the results above, we compared the combination to each compound of Esc, Cur and Her at the indicated optimal concentrations or dosages (Esc 11.6 μM, Cur 8.5 μM, and Hes 12.5 μM *in vitro* and Esc 337 mg·kg^−1^, Cur 60 mg·kg^−1^, and Hes 191 mg·kg^−1^ *in vivo*.) on LPS-induced the production of the inflammatory mediators and cytokines *in vitro*, and croton-oil induced mouse ear edema *in vivo*. The results showed that the combination of Esc, Cur and Her at the indicated optimal concentrations did not have any significant cytotoxic effects on Raw264.7 cells and inhibited the production of NO, PGE_2_, TNF-α, IL-1β and IL-6 *in vitro* and decreased ear edema *in vivo* significantly (Fig. 4).

## Discussion

*I. stolonifera* has been consumed for many years and touted for its anti-inflammatory properties. Relatively recent research has identified a number of compounds, isolated in n-butanol, that could be responsible for the anti-inflammatory properties of the herb. Despite their discovery and the implications for the treatment of inflammation, how these compounds interact, as well as their mechanisms of action are still unclear. Therefore, research into the synergistic potential between compounds as well as dosage optimization is a vital area of research.

Single herbs with multiple constituents can have multiple targets, suggesting that efficacy can be achieved by the synergistic and dynamic interactions among the multiple constituents. Overall quantitative analysis of drug combinations often involves multiple factors and multiple levels. Such studies mostly use orthogonal design^17^,^18^,^19^ and the orthogonal t value method^20^. These experiments can identify primary and secondary factors, but cannot be used to find the optimal concentrations of drugs in combination when there are many dosage levels. An improved uniform design method^21^,^22^,^23^,^24^,^25^,^26^ is available for multiple drugs combination analysis to determine optimal concentrations of drugs, especially the constituents of Chinese complex prescriptions^27^. This study represents the first attempt at applying the orthogonal design in order to identify primary and secondary interaction of compounds isolated from *I. stolonifera*. Initially, we sought to prove that synergistic effects existed between principal constituents at any ratio, by using the median-effect method, and then use the uniform design method to find the optimal concentrations and best efficacy of principal constituents for use in combination. Results of preliminary investigations indicated that synergistic interactions between principal constituents at any ratio may not be obvious in most situations. Thus we first identified the optimal concentrations of principal constituents by uniform design, and then using the median-effect method, we found that synergistic effects clearly exist between the principal constituents when they are used at the optimal concentrations.

Compounds isolated from *I. stolonifera* were found to inhibit LPS-induced PGE_2_ release, a widely used inflammation marker^28^,^29^. Collected data indicated that the inhibition of cyclooxygenase (COX)-influenced arachidonic acid metabolism may result in a subsequent reduction in PGE_2_ synthesis and inhibition of the inflammatory response. Therefore, the model of LPS-induced PGE_2_ release in Raw264.7 was adopted to evaluate the combined effect of compounds from *I. stolonifera*. Direct analysis and variance analysis were used to analyze the orthogonal experiment results^30^. The former is direct and easy to use, and has a low computing work load, but it cannot estimate experimental error. Namely, it can not distinguish whether the cause of experimental result variation is either due to variations in the tested factors or random. Analysis of variance can solve this problem. For analysis of variance, random error should be estimated and obtained from the blank column in the orthogonal chart. To reduce experiment times, the minimized sum of squares of deviations was chosen as an approximate evaluation. Results of both analyses indicated that the compounds that significantly affected release of PGE_2_ are Esc (*P* < 0.01), Cur (*P* < 0.01), and Hes (*P* < 0.05). Thus, they were identified to be the principal constituents from BE-IS. In uniform design experimental data processing, all factors should be used for regression analysis. The purpose of this is to obtain the optimal concentrations or dosages through partial derivatives. The expected best efficacy was found by response surface analysis, and at the optimal concentrations, the median-effect method was used to analyze the interaction among principal constituents. The median-effect method, namely Chou-Talalay combination index method^31^, is deduced by Chou and Talalay from the median-effect equation based on the law of mass action. As a mechanism-based, combined action analytical method, it has been one of the most commonly used methods. The results show that the principal constituents have synergistic effects when used at the optimal concentrations, suggesting that the anti-inflammatory effect of BE-IS is caused by synergistic actions of the individual compounds.

Data indicate the distinct abilities of the identified compounds to inhibit inflammatory mediators and cytokines. For example, Cur inhibits LPS-induced the production of NO and IL-1β most significantly, whereas Esc inhibits IL-6, but has no effect on LPS-induced the production of TNF-α and IL-1β. We speculate they probably act during different steps of inflammation, thus the combined use of these individual compounds can exert synergistic anti-inflammatory effects. We find that combined use of principal constituents from BE-IS can improve treatment efficacy while reducing the dosages. The verification results futher demonstrate that this combination is indeed synergistic, which could inhibit the production of inflammatory mediators and cytokines more significantly than sigle use of the compounds at the indicated optimal concentrations.

The three compounds (Esc, Cur and Her) exist in the highest quantity in the extract compared to the other bioactive compounds in the herb. Like the n-butanol extract, chloroform, acetic ether and aqueous extracts of *I. stolonifera* probably have the similar results that Esc, Cur and Her still have synergistic effects in the extracts. Though the three compounds show synergistic effects on the inhibiton of PGE_2_ release, they would not so sure have synergistic effects if NO, TNF-a, IL-1β or IL-6 levels were used as the parameter for the basis of the orthogonal design, uniform design and median effect analysis. In our study, the combination of Esc, Cur and Her at the indicated optimal concentration inhibits NO, TNF-a, IL-1β or IL-6 levels effectively. Based on the results, the effects of combining the three compounds are as good as using whole n-butanol extract. Obviously, the combination has more advantages, such as the convenient and abundant resource, and the certainty composition. We suggest that using the combination of the three compounds instead of whole herbal extract for its anti-inflammatory function. That is just why we study the compatibility and dose optimization of mutiple compounds from the herb.

## Conclusions

This study provides a new mode for compatibility analysis and concentration optimization of compound preparations of Chinese medicine. The mode includes screening principal constituents by orthogonal experiment, analyzing the optimal concentrations and best efficacy of principal constituents by uniform design, determining the synergistic effects between principal constituents by the median-effect method and investigating the mechanisms of these synergistic effects.

## Methods

### Animals

Male 4-week-old Kunming mice kept under SPF (specific-pathogen-free) conditions were obtained from the Laboratory Animal Center, Shantou University Medical College (Shantou, China). Animals were maintained in a room at a controlled temperature of 23 ± 2 °C and a fixed 12h light/dark cycle with free access to food and water. The total number of animals used in this study was 182 for the croton oil-induced mouse ear edema model. All studies involving animals are reported in accordance with the ARRIVE guidelines for reporting experiments involving animals^32^,^33^. All protocols involving animals were approved by the Institutional Animal Care and Use Committee of Shantou University Medical College.

### Cell culture and reagents

The experimental reagents purchased were as follows: Raw264.7 cell line from the Cell Bank of Chinese Academy of Sciences (Shanghai, China); LPS (*Escherichia coli*, 055:B5), DMSO, MTT, Sco and Cur from Sigma (St. Louis, MO, USA) and Esc, Umb and Hes from Alfa Aesar (Ward Hill, Massachusetts, USA). Cells were cultured in DMEM containing 10% FBS (Hyclone, Logan, UT, USA), 100 U·mL^−1^ penicillin and 100 μg·mL^−1^ streptomycin.

### MTT assay

The cytotoxicity of compounds from BE-IS was determined by MTT assay^34^. Raw264.7 cells (~1 × 10^5^ cells·mL^−1^) were plated onto 96-well culture plates and incubated overnight. The cells were untreated or treated with 1 μg·mL^−1^ LPS, 0.1% DMSO, BE-IS compounds (dissolved in 0.1% DMSO) or 1 h treatment of BE-IS compounds, followed by 1 μg·mL^−1^ LPS, and then incubated at 37 °C under a humidified atmosphere with 5% CO_2_ for 24 h. Cells were then incubated with MTT for 4 h, followed by the addition of 150 μL DMSO per well. After solubilizing completely, cytotoxicity was determined by measuring the OD at 550 nm using a microplate reader.

### Measurement of PGE_2_, NO and cytokines

Measurement of cytokines, NO and PGE_2_ was performed as previously described^7^. Briefly, Raw264.7 cells were plated ~2 × 10^5^ cells·mL^−1^ per well in 24-well culture plates. Twenty-four hours later, cells were treated with BE-IS compounds, individually or combined, for one hour, before stimulation with 1 μg·mL^−1^ LPS for 24 h. The supernatant was collected and the amount of secreted PGE_2_ and cytokines was measured by specific ELISA kits (PGE_2_, Enzo Life Sciences Inc., NY, USA; TNF-α, IL-1β and IL-6, Boster Bioengineering Ltd, Wuhan, China) according to the manufacturer’s instructions. The level of NO in the culture supernatant was measured using an NO assay kit (Jiancheng Bioengineering Institute, Nanjing, China) according to the manufacturer’s instructions.

To study their dose-efficacy relationship of single compounds on inhibiting PGE_2_ release, the concentration-effect relationship of each compound from BE-IS was fitted to the Hill equation y = E_max_ × x^n^/(k^n^ + x^n^), using Origin7.5 software.

### Orthogonal experimental design

Based on the effective concentration range of compounds from BE-IS, the L_16_(4^5^) orthogonal design table^17^ was used to design an experiment with 4 levels per compound and a total of 16 combinations. Inhibition of LPS-induced PGE_2_ release was used as an indicator to evaluate the efficacy. Through direct analysis and variance analysis, the effect of each compound on the efficacy of the tested combinations was determined to screen the principal constituents in BE-IS.

### Uniform experimental design

Based on the results from orthogonal experimental design, the U_7_(7^3^) uniform design table^23^,^27^ was used to design an experiment with 7 different combinations. Inhibition of LPS-induced PGE_2_ release was used as an indicator to evaluate the efficacy. A multivariate regression equation was established using Excel Solver tool, and optimal concentrations, as well as efficacy of combined use of principal constituents from BE-IS were determined by the response surface method.

### Median-effect method

Based on the results from uniform experimental design, the proportion for the combined use of the principal constituents was set. The median-effect method was used to analyze the interaction among principal constituents by using the following equation: Fa/Fu = (D/D_50_)^m^, where Fa denotes efficacy, Fu = 1-Fa, D indicates drug dosage, m is the parameter, and D_50_ refers to the 50% efficacy dosage. In order to account for the variable D_50_ of the drugs and m, the logarithm was taken as: log (Fa/Fu) = mlogD–mlogD_50_ (1). For each drug, dosage D and the corresponding inhibition rates Fa were determined from the experiment. The D_50_ and Fa were determined from equation (1) by doubling the logarithm coordinates and performing a linear regression analysis

Assuming the test drugs were Drug 1, Drug 2, and their combination (Drug 1, 2), when inhibition rate Fa = a%, the required dosage (Da)1, (Da)2, and (Da)1, 2 could be calculated from equation (1). Assuming the ratio of drug 1 to drug 2 in Drug 1, 2 was a:b, the dosage of drug 1 and drug 2 in (Da)1, 2 can be calculated by: (da)1 = (Da)1, 2*a/(a + b) (2); (da)2 = (Da)1, 2*b/(a + b) (3). The combination index (CI), which indicates the efficacy of drugs used in combination, can be obtained based on the median-effect method: (CI)a = (da)1/(Da)1 + (da)2/(Da)2 + k*(da)1(da)2/(Da)1(Da)2 (4), where k refers to the parameter for combined action. When the effects of two drugs are not independent, k = 0. When the effects of two drugs are independent, k = 1. Therefore, a CI < 1 indicates synergy, CI = 1 indicates additivity, and CI > 1 indicates an antagonistic effect between the two drugs.

### Croton oil-induced ear edema model

Croton oil-induced ear edema was performed as described previously^35^,^36^. Mice were divided randomly into the following eight groups (12 mice per group) for oral gavage treatment once a day for 5 days: control group (0.5% CMC-Na, 0.2 ml) and seven treated groups (the combination of different doses of Esc, Cur and Hes). One hour after the last drug administration, ear edema was induced by topical application of 1% croton oil in mixture solvent (v/v, croton oil: ethanol: pyridine: ethyl ether = 1:10:20:69, 100 ul) on the outer and inner surfaces of the right ear of each mouse. The left ear remained untreated and served as a control. The mice were killed by cervical dislocation 2 h after the application of the irritant. An ear disk, 8.0 mm in diameter, was punched out and weighed. The weight difference between the left and the right ear disk of the same animal was evaluated as the extent of edema. The inhibition percentage was calculated by the following equation:


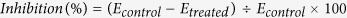


where *E*_control_ and *E*_treated_ is the extent of edema from the control group and treated groups.

### Uniform design *in vivo* experiment

The U_7_(7^3^) uniform design table was used to design an experiment with 7 different combinations. Inhibition of croton oil-induced edema in the mouse ear was used as an indicator to evaluate the efficacy. A multivariate regression equation was established, and optimal concentrations as well as efficacy of combined use of principal constituents from BE-IS, were determined with Excel Solver tool.

### Verification experiments

According to the results above, the optimal concentrations or dosages of principal constituents were: Esc 11.6 μM, Cur 8.5 μM, and Hes 12.5 μM *in vitro* and Esc 337 mg·kg^−1^, Cur 60 mg·kg^−1^, and Hes 191 mg·kg^−1^ *in vivo*. To prove the results, the experiments below were designed. MTT assay was performed to determine whether the combination of Esc 11.6 μM, Cur 8.5 μM, and Hes 12.5 μM affects viability of Raw264.7 cells. In LPS-induced Raw264.7 cells, NO assay kits and specific ELISA kits were used to determine the levels of NO, PGE_2_, TNF-α, IL-1β and IL-6 after treatment with the combination and each compound of Esc, Cur and Her at the indicated optimal concentrations. Croton oil-induced mouse ear edema was performed to determine that the inhibition of ear edema after treatment with the combination and each compound of Esc, Cur and Her at the indicated optimal dosages.

### Statistical analysis

The experimental data were analyzed with the SPSS 17.0 software and presented as the mean ± standard error (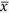 ± S.E.). Comparison between groups was performed using one-way ANOVA, and results were considered statistically significant when P < 0.05.

## Additional Information

**How to cite this article**: Cai, C. *et al.* Synergistic Effect of Compounds from a Chinese Herb: Compatibility and Dose Optimization of Compounds from N-Butanol Extract of Ipomoea stolonifera. *Sci. Rep.* **6**, 27014; doi: 10.1038/srep27014 (2016).

## Figures and Tables

**Table 1:** Related parameters of the Hill equation.

Compounds	E_max_	k (IC_50_)	n	R^2^	R
Sco	99.06%	15.99	1.4126	0.99763	0.99881
Umb	50.21%	70.20	1.35607	0.99662	0.99831
Esc	80.15%	9.07	2.89305	0.99058	0.99528
Hes	98.23%	12.80	1.46786	0.98996	0.99497
Cur	112.25%	3.57	0.74563	0.99212	0.99605

**Table 2:** L_16_(4^5^) orthogonal experiment chart, results, direct analysis and variance analysis.

NO.	Sco (μM)	Umb (μM)	Esc (μM)	Hes (μM)	Cur (μM)	Inhibition of PGE_2_ release (%)
1	5	5	1	1	1	11.90
2	5	10	4	4	3	50.05
3	5	15	7	7	5	73.69
4	5	20	10	10	7	93.31
5	10	5	4	7	7	81.27
6	10	10	1	10	5	50.56
7	10	15	10	1	3	70.88
8	10	20	7	4	1	42.52
9	15	5	7	10	3	78.64
10	15	10	10	7	1	65.31
11	15	15	1	4	7	39.32
12	15	20	4	1	5	52.91
13	20	5	10	4	5	81.19
14	20	10	7	1	7	83.78
15	20	15	4	10	1	46.20
16	20	20	1	7	3	34.50
K1	228.95	253.00	136.29	219.48	165.92	
K2	245.24	249.71	230.43	213.08	234.08	
K3	236.19	230.09	278.63	254.77	258.35	
K4	245.67	223.24	310.69	268.72	297.69	
avK1	57.24	63.25	34.07	54.87	41.48	
avK2	61.31	62.43	57.61	53.27	58.52	
avK3	59.05	57.52	69.66	63.69	64.59	
avK4	61.42	55.81	77.67	67.18	74.42	
R	4.18	7.44	43.60	13.91	32.94	
**Analysis of variance**						
**Variation source**	**Sum of squares of deviations**	**Degree of freedom**	**Mean square**	***F*** **value**	***P*** **value**	
Esc	4333.47	3	1444.49	90.15	<0.01	
Cur	2295.98	3	765.33	47.76	<0.01	
Hes	546.17	3	182.06	11.36	<0.05	
Umb	159.58	3	53.19	3.32	>0.10	
Sco	48.07	3	16.02			

F_0.01_(3, 3) = 29.46, F_0.05_(3, 3) = 9.28, F_0.10_(3, 3) = 5.36.

**Table 3:** U_7_(7^3^) uniform experiment chart, results and replication experiment *in vitro.*

NO.	Esc (μM)	Cur (μM)	Hes (μM)	Y (Inhibition of PGE_2_ release, %)
1	3	3	7	42.20
2	5	5	13	67.49
3	7	7	5	72.37
4	9	2	11	68.01
5	11	4	3	58.44
6	13	6	9	83.76
7	15	8	15	91.55
**Replication experiment**				
**Group**	**PGE**_**2**_ **(pg·mL**^**−1**^)	**Actual inhibition (%)**	**Fitted inhibition (%)**	
Normal	243.00			
LPS	8127.74			
Expected optimal	402.95	97.97	99.77	
Experiment optimal	537.15	96.27	91.54	

**Table 4:** Drug concentration and corresponding inhibition of PGE_2_ release.

Drug	Dosage/D (μM) and corresponding inhibition rate/Fa (%)					
Esc	Fa	5.92	9.42	17.87	30.87	56.52
	D	0.725	1.45	2.9	5.8	11.6
Cur	Fa	9.89	23.59	39.63	55.48	71.08
	D	0.53125	1.0625	2.125	4.25	8.5
Hes	Fa	4.47	11.23	18.63	28.96	49.37
	D	0.78125	1.5625	3.125	6.25	12.5
E+C*	Fa	18.64	36.89	56.47	69.63	83.24
	D	1.25625	2.5125	5.025	10.05	20.1
E+H*	Fa	10.53	17.38	31.32	48.29	72.32
	D	1.50625	3.0125	6.025	12.05	24.1
C+H*	Fa	15.89	29.33	49.38	66.21	79.48
	D	1.3125	2.625	5.25	10.5	21

^*^E+C represent combination of Esc and Cur; E+H represent combination of Esc and Hes; C+H represent combination of Cur and Hes.

**Table 5:** Coefficient correlation r, parameter m, median effective dosage D_50_ and Combination Index (CI).

	Esc	Cur	Hes	E + C*	E + H*	C + H*		
r	0.9917	0.9950	0.9949	0.9967	0.9952	0.9989		
m	1.0840	1.0983	1.0451	1.0848	1.1095	1.0955		
mlogD_50_	1.1178	0.5929	1.1709	0.7013	1.1774	0.8361		
D_50_	10.7444	3.4660	13.1938	4.4308	11.5133	5.7971		
**Combination Index (CI)**								
**Fa(%)**	**10**	**30**	**50**	**70**	**80**	**85**	**90**	**95**
E+C*	0.7656	0.7736	0.7786	0.7837	0.7869	0.7890	0.7919	0.7964
E+H*	1.0517	0.9995	0.9684	0.9384	0.9199	0.9082	0.8929	0.8689
C+H*	0.9616	0.9471	0.9385	0.9303	0.9252	0.9220	0.9179	0.9114

^*^E+C represent combination of Esc and Cur; E+H represent combination of Esc and Hes; C+H represent combination of Cur and Hes.

**Table 6:** U_7_(7^3^) uniform experiment chart, results and replication experiment *in vivo.*

NO.	Esc (mg·kg^−1^)	Cur (mg·kg^−1^)	Hes (mg·kg^−1^)	Y (Inhibition of ear edema, %)
1	60	50	140	24.32
2	100	70	260	28.36
3	140	90	100	23.32
4	180	40	220	44.48
5	220	60	60	47.04
6	260	80	180	67.12
7	300	100	300	57.04
**Replication experiment**				
**Group**	**Swelling degree (mg)**	**Actual inhibition rate (%)**	**Fitting inhibition rate (%)**	
Model	14.35			
Expected optimal	3.56	75.19	99.63	
Experimental optimal	4.92	65.71	67.12	
